# Dietary trends and obesity in Saudi Arabia

**DOI:** 10.3389/fpubh.2023.1326418

**Published:** 2024-01-11

**Authors:** Noara Alhusseini, Nawra Alsinan, Shahad Almutahhar, Majd Khader, Rawand Tamimi, Mazin Ibrahim Elsarrag, Rabah Warar, Sara Alnasser, Majed Ramadan, Aamir Omair, Sihem Aouabdi, Rimah Saleem, Alaa Alabadi-Bierman

**Affiliations:** ^1^College of Medicine, Alfaisal University, Riyadh, Saudi Arabia; ^2^Prince Sultan Military Medical City, Riyadh, Saudi Arabia; ^3^King Abdullah International Medical Research Center, Jeddah, Saudi Arabia; ^4^King Saud Bin Abdulaziz University for Health Sciences, Jeddah, Saudi Arabia; ^5^King Saud bin Abdulaziz University for Health Sciences, Riyadh, Saudi Arabia; ^6^Ministry of National Guard, King Abdulaziz Medical City, Jeddah, Saudi Arabia; ^7^School of Public Health, Loma Linda University, Loma Linda, CA, United States; ^8^University of California, Riverside, Riverside, CA, United States

**Keywords:** diet, Saudi Arabia, dietary trends, ketogenic diet, intermittent fasting, gluten-free diet, calorie restriction

## Abstract

**Introduction:**

Dietary habits in Saudi Arabia have been shifting toward the Western diet, which is high in fat, salt, and sugar, leading to a high obesity rate. Different dietary strategies such as the Ketogenic Diet (KD), Intermittent Fasting (IF), Gluten Free Diet (GFD), and Calorie Restriction Diet (CRD) have shown an influential role in weight loss. This study aimed to compare trending diets and correlate different types of diet with obesity and lifestyle among adults in Saudi Arabia.

**Methods:**

A cross-sectional study was performed on Saudis and non-Saudis over 18 years old. We used convenience sampling, an online questionnaire distributed via social media channels, including WhatsApp, LinkedIn, and Twitter. SPSS 28 software was applied for data analysis. The chi-square test was used to determine associations between different variables. Statistical significance was considered at a value of *p* less than 0.05.

**Results:**

Most participants were females residing in the Eastern and Central regions of Saudi Arabia. Although most do not follow any dietary plan, they exhibited acceptable exercise and lifestyle. The minority of the study population followed different types of diet plans, such as KD, IF, and GFD. The purpose of most of the participants who have used these strategies was for weight loss but failed to sustain the dietary plan for more than 1 month.

**Conclusion:**

Obesity remains a challenging issue in Saudi Arabia. Adherence to dietary regimes could help in controlling obesity. Increasing the awareness of the benefits of each dietary plan for health, choosing the appropriate one, and sustaining a balanced nutrition pattern.

## Introduction

Obesity is a medical condition characterized by the excessive accumulation of body fat to the extent that it may have a negative impact on health. It is often determined by body mass index (BMI), which is a measure of body fat based on a person’s weight and height. BMI is calculated by dividing a person’s weight in kilograms by the square of their height in meters. The World Health Organization (WHO) classifies adults’ BMI into the following categories: Underweight: BMI less than 18.5 kg/m2, Normal weight: BMI 18.5 to 24.9, kg/m2 Overweight: BMI 25 to 29.9 kg/m2, Obesity (Class 1): BMI 30 to 34.9 kg/m2, Obesity (Class 2): BMI 35 to 39.9 kg/m2, Obesity (Class 3 - severe or morbid obesity): BMI of 40 or higher kg/m2 (1). Sedentary behaviors and unhealthy dietary patterns are significant risk factors for obesity. Obesity is associated with an increased risk of type 2 diabetes, hypertension, and cardiovascular disorders. (2, 3). Addressing obesity often involves lifestyle modifications, including adopting a healthy diet, increasing physical activity, and, in some cases, medical interventions. (4, 5).

Obesity is a significant health concern worldwide, and Saudi Arabia is no exception. The prevalence of obesity has been on the rise in Saudi Arabia over the past few decades, with several factors contributing to this trend. Approximately 20% of Saudis are obese, with the majority being older adult married women (6). Urbanization and economic development in Saudi Arabia have led to changes in lifestyle, including increased sedentary behavior and a shift toward more energy-dense diets. These changes, often associated with urban living, have contributed to the obesity epidemic. Obesity rates will likely continue escalating due to the increased dependence on fast food chains, which are high in fat, salt, and sugar (7–9). More than 15,000 bariatric surgeries are performed annually in Saudi Arabia, costing up to 40,000 Saudi Riyals (SAR) for each surgery. Such high obesity rates and chronic conditions cause a burden on the public health and healthcare systems (10). Healthy diet and exercise are cost-effective and positively impact human weight loss and well-being (11). Several dietary strategies have shown a role in weight loss, such as a ketogenetic diet (KD), Intermittent Fasting (IF), Gluten-Free Diet (GFD), and the Calorie Restriction Diet (CRD).

The KD, is a popular diet that has been used since the 1920s to treat epilepsy. It is often referred to as the keto diet, which is a low-carbohydrate, high-fat diet designed to induce a state of ketosis in the body. Ketosis is a metabolic state where the body shifts from using glucose as its primary source of energy to relying on ketones, which are molecules produced from the breakdown of fats. This change in fuel source has various effects on the body, including increased fat burning. The macronutrient distribution in a standard ketogenic diet typically involves three components. High Fat: Approximately 70–75% of total daily calories come from fats. This includes sources like avocados, nuts, seeds, oils, and fatty cuts of meat. Moderate Protein: About 20–25% of calories come from protein. Protein sources include meat, poultry, fish, eggs, and dairy. Low Carbohydrate: Carbohydrates are severely restricted, making up only about 5–10% of total daily calories. This often limits daily carbohydrate intake to around 20–50 grams, depending on individual needs (12). The initial weight loss on a ketogenic diet is often rapid due to water loss associated with glycogen depletion. This can be motivating for some individuals, but it’s essential to differentiate between water weight and actual fat loss. Some people experience reduced appetite when following a ketogenic diet, which can contribute to weight loss. Recent studies using low-carbohydrate, high-fat diets, such as KD, show promising outcomes on weight loss, reversing the symptoms of metabolic syndrome, lowering or eliminating the need for insulin in type II diabetes mellitus, reducing inflammation, improving epigenetic profiles, changing the microbiome, enhancing lipid profiles, and assisting cancer treatment (13).

Another popular diet is intermittent fasting (IF), which relies on consuming food for eating for a specific timeframe followed by fasting for the rest of the day (14). Intermittent fasting (IF) is an eating pattern that cycles between periods of eating and fasting. There are several variations of intermittent fasting, but common methods include the 16/8 method (fasting for 16 h and eating within an 8-h window), the 5:2 method (eating normally for 5 days and significantly reducing calorie intake on two non-consecutive days), and the eat-stop-eat method (24-h fasts once or twice a week). Intermittent fasting can lead to various metabolic changes in the body and some of these changes may include insulin sensitivity. Improved sensitivity means that cells are better able to respond to insulin, which can be beneficial for managing blood sugar levels. Moreover, fasting periods can lead to an increase in the breakdown of fat stores for energy, promoting the process of lipolysis. This can contribute to weight loss and fat reduction. In addition, intermittent fasting may influence lipid profiles, including reducing triglyceride levels and improving cholesterol levels, which could contribute to cardiovascular health (15).

Fasting improves physical and mental performance by increasing metabolic and nervous system activities (16). IF is an effective dietary plan for weight loss and reversing metabolic parameters such as cholesterol, triglycerides, and oxidative stress markers (14, 17).

On the other hand, the GFD constricts the consumption of wheat, barley, and rye sources. This diet relies on gluten-free, food such as eggs, fruits, vegetables, meat, fish, legumes, dairy products, and wheat-based food manufactured without gluten (18). GFD is usually medicated for patients with celiac disease as it improves clinical manifestation and blood parameters (19, 20). Although people who are not suffering from celiac disease use GFD, they are susceptible to nutritional deficiencies such as iron and vitamin B. Adopting a gluten-free diet may impact nutrient intake. Whole grains containing gluten, such as wheat, provide essential nutrients like fiber, B vitamins, and minerals. When removing gluten-containing grains, individuals must ensure they get these nutrients from alternative sources. Some people may experience weight changes when they switch to a gluten-free diet. Various factors, including the types of gluten-free foods chosen, can influence this. Some gluten-free products may be higher in calories, sugar, and fat compared to their gluten-containing counterparts (21).

In addition, the calorie restriction diet (CRD) entails a sustained reduction in daily caloric intake of roughly 25–30% from the average calorie intake (22). The CRD is effective in terms of weight loss and disease risk (23). The most immediate and noticeable effect of a calorie-restricted diet is often weight loss. When the body receives fewer calories than it needs for its daily activities, it starts utilizing stored energy, primarily in the form of glycogen and fat, leading to a reduction in body weight. The Calorie Restriction Society reports that participants who adhere to a self-imposed CRD program have more extended life expectancies. Moreover, caloric restriction may enhance insulin sensitivity, which is the body’s ability to respond to insulin and regulate blood sugar levels. Improved insulin sensitivity is associated with better metabolic health and a reduced risk of type 2 diabetes. (24).

Therefore, this study aimed to describe different diet trends and assess which type of diet correlates with obesity among adults residing in Saudi Arabia. This included exploring the prevalence of KD, IF, GFD, and CRD alongside the correlation between diet trends and obesity and assessing predictors of diet trends among the Saudi population.

## Materials and methods

This cross-sectional study focused on different diets used among residents of Saudi Arabia. Convenience sampling was used, and the minimum sample size requirement was (*n* = 384) by a confidence interval of 95% and a 5% margin of error with an expected outcome response of 50%. However, the authors collected (*n* = 608) responses. The inclusion criteria were adults above 18 years old, including Saudi citizens and Non-Saudi residents at the time of the data collection.

An online survey was distributed via social media channels such as WhatsApp, LinkedIn, Facebook, and Twitter in Arabic and English. Clinical nutrition experts and an official translator achieved face and content validity. The survey included two sections; section 1 comprised demographic questions such as age, gender, nationality, marital status, employment status, educational level, income and residence. Section 2 covered dietary habits-related questions which was categorized into KD, IF, GFD, CRD, and no dietary restrictions. In addition, the mean body mass index (BMI) values were calculated for each group. The SPSS version 28 statistical software was used for data analysis. Frequencies and percentages were applied for data representation. In addition, the Pearson chi-square (*χ*^2^) and logistic regression were used to evaluate the potential association between dietary trends and obesity, and potential confounding variables were added to the multivariable model with odds ratios (ORs) and 95% confidence intervals (CIs) reported.

No identifying information, such as names or emails, was collected in this study, and data access was restricted to the authors only. Participation was voluntary, and the consent form was provided to all participants before participation and participants were able to withdraw at any time. The study was conducted upon approval from the Institutional Review Board (IRB) at King Abdullah International Medical Research Center under the reference number NRJ22J/288/11.

## Results

The total of 608 participants who responded to the survey were included in this study. We initially assessed the demographic characteristics of the respondents, such as age, gender, nationality, marital status and employment status, employment status, type of qualification, income, and residence. Most of the participants in this study were less than 45 years old, where females (66%) and Saudis (76%) represented the majority of the study population. More than half were married (58%), and the highest proportion were employed (39%). Most participants hold bachelor’s degrees or diplomas, and 32% have no income. Interestingly, the highest number of participants were residing in the eastern region (45%), followed by the central region (44%) of Saudi Arabia (Table 1).

**Table 1:** Demographic characteristics of the respondents.

		*n*	%
Age	18–29 years	233	38%
	30–44 years	201	33%
	45–64 years	156	26%
	65+ years	18	3%
Gender	Male	205	34%
	Female	403	66%
Nationality	Saudi	462	76%
	Non-Saudi	146	24%
Marital status	Single	232	38%
	Married	351	58%
	Divorced	17	3%
	Widowed	8	1%
Employment status	Employed	239	39%
	Unemployed	135	22%
	Retired	58	10%
	Self-employed	19	3%
	Student living with family	132	22%
	Student living away from home	25	4%
Education level	High school or less	130	21%
	Bachelor’s degree/Diploma	401	66%
	Higher degree (Masters, PhD or equivalent)	77	13%
Income	I do not have an income	195	32%
	<10,000 SR	129	21%
	10,000 to <20,000 SR	112	18%
	20,000+ SR	78	13%
	I prefer not to say	94	15%
Region	Eastern	272	45%
	Western	60	10%
	Central	270	44%
	Northern	3	0%
	Southern	3	0%

The respondents’ BMI was 26.7 ± 5.4 kg/m^2^, with a range of 14.5 to 48.6 kg/m^2^. Next, we evaluated exercise and dietary habits, where 30% of the respondents did not exercise, and 45% often followed a balanced diet. In addition, 63% of the participants have fruits and 57% vegetables, about 1–3 times per week. More than half of the study population eat from restaurants (61%), have 2 meals a day (51%), and their last meal is between 7 and 10 pm (61%) (Table 2). Interestingly, 75% of the respondents do not follow any diet plan, while the minority follow intermittent fasting (10%), low-fat diet (8%), and low carbohydrate or Atkins diet (4%) (Table 3).

**Table 2:** Exercise and dietary habits as self-reported by the respondents.

		*n*	%
How often do you exercise for 30 min or more?	I do not exercise at all	184	30%
	Mild (1–2 times per month)	190	31%
	Moderate (2–3 times per week)	170	28%
	Extreme (4–6 times per week)	64	11%
Do you eat a healthy balanced meal (carbohydrates, protein, and fat)?	No	90	15%
	Yes	231	38%
	Sometimes	287	47%
How many servings of fruits do you eat per week?	0	90	15%
	1–3	385	63%
	4 or more	133	22%
How many servings of vegetables do you eat per week?	0	48	8%
	1–3	345	57%
	4 or more	215	35%
How often do you eat from restaurants?	I do not eat from restaurants	23	4%
	Monthly	123	20%
	Weekly	370	61%
	Daily	92	15%
How many meal(s) do you eat per day?	1	31	5%
	2	310	51%
	3	230	38%
	>3	37	6%
What time is your last meal?	Before 7 pm	96	16%
	7–10 pm	369	61%
	After 10 pm	143	24%

**Table 3:** Type of diet followed by the respondents.

		*n*	%
Which diet do you currently follow?	No specific diet	456	75%
	Intermittent fasting	59	10%
	Low fat diet	48	8%
	Low carb or atkins diet	27	4%
	DASH diet	4	1%
	Ketogenic diet	3	0%
	Mediterranean diet	3	0%
	Vegetarian or vegan diet	2	0%
	Restricted calorie diet	0	0%
	Other	6	1%

The majority of this population tried the KD (85%), learned about it from the internet (54%), and primarily followed it for weight loss (84%). However, 52% of the participants have been on the KD for less than a month and tried it once (59%) (Table 4). In addition, 63% of the participants have tried IF and learned about it from the internet (56%). The majority also followed IF for weight loss (75%) and applied this plan for less than 1 month (39%). The fasting pattern was variable as 23% involved 16 h; others used 14 (22%) and 12 h, representing a percentage of 22 and 20%, respectively (Table 5).

**Table 4:** Respondents following KD.

		*n*	%
Have you tried the ketogenic diet?	No	515	85%
	Yes	93	15%
How did you learn about keto diet?	Internet	50	54%
	Books	0	0%
	Medical professional	9	10%
	Family member or friend	33	35%
	Community group	1	1%
	Other	0	0%
Why did you start the keto diet?	To improve chronic health condition	6	6%
	For weight loss	78	84%
	To enhance athletic performance	2	2%
	Trending diet	2	2%
	No specific reason - just curious	5	5%
	Other	0	0%
How long have you been on the keto diet?	< 1 month	48	52%
	1–3 months	25	27%
	4–6 months	10	11%
	7–12 months	3	3%
	>12 months	7	8%
How many times have you followed the keto diet?	Once	55	59%
	Twice	24	26%
	Thrice	6	6%
	Many times	8	9%

**Table 5:** Respondents following IF.

		*n*	%
Have you tried intermitting fasting?	No	380	63%
	Yes	228	38%
How did you learn about intermittent fasting?	Internet	128	56%
	Books	2	1%
	Medical professional	18	8%
	Family member or friend	64	28%
	Community group	7	3%
	Other	9	4%
Why did you start the intermitting fasting diet?	To improve chronic health condition	17	8%
	For weight loss	168	75%
	To enhance athletic performance	6	3%
	Trending diet	6	3%
	No specific reason - just curious	22	10%
	Other	5	2%
How long did you practice intermittent fasting for?	< 1 month	88	39%
	1–3 months	53	23%
	4–6 months	25	11%
	7–12 months	16	7%
	>12 months	46	20%
What was the fasting pattern you followed?	12 h fasting	51	22%
	14 h fasting	52	23%
	16 h fasting	75	33%
	18 h fasting	13	6%
	2 days per week	15	7%
	Rotating fasting	19	8%
	Other	3	1%

Furthermore, 33% of the participants have been on CRD, mostly learned about this diet plan from the internet (45%) and used it for weight loss (85%). This majority of this group either used this dietary strategy for less than 1 month or more than 12 months (Table 6). However, the GFD was the highest percentage of dietary plans the respondents applied (95%). Some participants learned about this dietary plan from the Internet (39%), whereas others learned about it from medical professionals (29%) or family members and friends (25%). Furthermore, the majority applied this dietary plan for different reasons, such as weight loss (25%), celiac disease (11%), or skin allergy (14%). However, 37% used it for less than 1 month, 29% for more than 12 months, while others used it between 1 and 12 months (Table 7).

**Table 6:** Respondents following CRD.

		*n*	%
Have you tried calorie restriction diet?	No	409	67%
	Yes	199	33%
How did you learn about calorie restriction diet?	Internet	89	45%
	Books	6	3%
	Medical professional	53	27%
	Family member or friend	41	21%
	Community group	9	5%
	Other	1	1%
Why did you start Calorie Restriction diet?	To improve chronic health condition	13	7%
	For weight loss	166	83%
	To enhance athletic performance	14	7%
	Trending diet	3	2%
	No specific reason - just curious	3	2%
	Other	0	0%
How long did you practice the calorie restricted diet?	< 1 month	48	24%
	1–3 months	61	31%
	4–6 months	24	12%
	7–12 months	20	10%
	>12 months	46	23%

**Table 7:** Respondents following the GFD.

		*n*	%
Have you tried gluten-free diet?	No	580	95%
	Yes	28	5%
How did you hear about the gluten-free diet?	Internet	11	39%
	Books	0	0%
	Medical professional	8	29%
	Family member or friend	7	25%
	Community group	2	7%
	Other	0	0%
Why did you start the gluten free diet?	I get unwell when I eat foods containing gluten	0	0%
	I think I have celiac disease but have not tested for it	0	0%
	I think I have gluten intolerance / sensitivity but have not tested for it	7	25%
	I am diagnosed with celiac disease	3	11%
	To improve energy levels	0	0%
	To lose weight	7	25%
	My friends/family are on it	0	0%
	It is cool to be on a diet	0	0%
	To be healthier	0	0%
	I have food/skin allergy	4	14%
	Personal beliefs	0	0%
	Other	7	25%
How long did you practice the gluten-free diet?	< 1 month	10	36%
	1–3 months	4	14%
	4–6 months	3	11%
	7–12 months	3	11%
	>12 months	8	29%

We then investigated the association between different types of diet with demographic variables (data are provided in the supplementary material) and eating habits. Age and chronic diseases were highly associated with any diet (*p* = 0.008) (Supplementary Table S1). In addition, exercise and consumption of fruits and vegetables were tightly correlated with any dietary plan (*p* < 0.001 and *p* = 0.011, respectively) (Table 8). Next, we investigated the association between IF, low carbohydrate, or low-fat diet with demographic variables and eating habits. Results showed no significant association between IF and demographic variables (Supplementary Table S2). However, exercise (*p* = 0.001), balanced meals (*p* = 0.012), number of meals per day (*p* = 0.006), and the time of participant’s last meal significantly differed between participants following IF and those who did not follow any dietary plan (Table 9). On the other hand, there was no significant association between demographic variables and low carbohydrate or low-fat diet, except for chronic diseases (*p* = 0.017) (Table S3). In contrast, significant associations existed between exercise (*p* < 0.001), balanced meals (*p* < 0.001), and consuming fruits (*p* = 0.049) and vegetables (*p* = 0.036) with a low carbohydrate or low-fat diet (Table 10).

**Table 8:** Association of eating habits with following any diet following any diet.

		Follow any diet				
		Yes		No		
		*n*	%	*n*	%	Value of *p*
How often do you exercise for 30 min or more?	I do not exercise at all	18	12%	166	36%	
	Mild (1–2 times per month)	45	30%	145	32%	<0.001*
	Moderate (2–3 times per week)	59	39%	111	24%	
	Extreme (4–6 times per week)	30	20%	34	7%	
Do you eat a healthy balanced meal (carbohydrates, protein, and fat)?	No	7	5%	83	18%	
	Yes	84	55%	147	32%	<0.001*
	Sometimes	61	40%	226	50%	
How many servings of fruits do you eat per week?	0	14	9%	76	17%	
	1–3	99	65%	286	63%	0.06
	4 or more	39	26%	94	21%	
How many servings of vegetables do you eat per week?	0	9	6%	39	9%	
	1–3	74	49%	271	59%	0.011*
	4 or more	69	45%	146	32%	
How often do you eat from restaurants?	I do not eat from restaurants	8	5%	15	3%	
	Monthly	37	24%	86	19%	0.06
	Weekly	93	61%	277	61%	
	Daily	14	9%	78	17%	
How many meal(s) do you eat per day?	1	3	2%	28	6%	
	2	82	54%	228	50%	0.14
	3	55	36%	175	38%	
	>3	12	8%	25	5%	
What time is your last meal?	Before 7 pm	35	23%	61	13%	
	7–10 pm	93	61%	276	61%	0.003*
	After 10 pm	24	16%	119	26%	

Bold means less than 0.05, *means significant values.

**Table 9:** Association of eating habits with following IF diet.

		Intermittent fasting diet				
		Yes (*n* = 59)		No (*n* = 549)		
		*n*	%	*n*	%	Value of *p*
How often do you exercise for 30 min or more?	I do not exercise at all	5	8%	179	33%	**0.001** ^*^
	Mild (1–2 times per month)	25	42%	165	30%	
	Moderate (2–3 times per week)	23	39%	147	27%	
	Extreme (4–6 times per week)	6	10%	58	11%	
Do you eat a healthy balanced meal?	No	1	2%	89	16%	**0.012** ^*^
	Yes	26	44%	205	37%	
	Sometimes	32	54%	255	46%	
How many servings of fruits do you eat per week?	0	5	8%	85	15%	0.15
	1–3	44	75%	341	62%	
	4 or more	10	17%	123	22%	
How many servings of vegetables do you eat per week?	0	5	8%	43	8%	0.45
	1–3	29	49%	316	58%	
	4 or more	25	42%	190	35%	
How often do you eat from restaurants?	I do not eat from restaurants	4	7%	19	3%	0.11
	Monthly	17	29%	106	19%	
	Weekly	33	56%	337	61%	
	Daily	5	8%	87	16%	
How many meal(s) do you eat per day?	1–2	43	73%	298	54%	**0.006**
	>3	16	27%	251	46%	
What time is your last meal?	Before 7 pm	19	32%	77	14%	**<0.001** ^*^
	7–10 pm	34	58%	335	61%	
	After 10 pm	6	10%	137	25%	

Bold means less than 0.05, *means significant values.

**Table 10:** Association of eating habits with following low carb or low-fat diet.

		Low Carb or Low Fat diet				
		Yes (*n* = 75)		No (*n* = 533)		
		*n*	Col %	*n*	Col %	Value of *p*
How often do you exercise for 30 min or more?	I do not exercise at all	11	15%	173	32%	**<0.001** ^*^
	Mild (1–2 times per month)	13	17%	177	33%	
	Moderate (2–3 times per week)	32	43%	138	26%	
	Extreme (4–6 times per week)	19	25%	45	8%	
Do you eat a healthy balanced meal?	No	3	4%	87	16%	**<0.001** ^*^
	Yes	48	64%	183	34%	
	Sometimes	24	32%	263	49%	
How many servings of fruits do you eat per week?	0	7	9%	83	16%	**0.049** ^*^
	1–3	44	59%	341	64%	
	4 or more	24	32%	109	20%	
How many servings of vegetables do you eat per week?	0	3	4%	45	8%	**0.036** ^*^
	1–3	36	48%	309	58%	
	4 or more	36	48%	179	34%	
How often do you eat from restaurants?	I do not eat from restaurants	4	5%	19	4%	0.61
	Monthly	15	20%	108	20%	
	Weekly	48	64%	322	60%	
	Daily	8	11%	84	16%	
How many meal(s) do you eat per day?	1–2	3	4%	304	57%	0.21
	>3	38	51%	229	43%	
What time is your last meal?	Before 7 pm	13	17%	83	16%	0.26
	7–10 pm	50	67%	319	60%	
	After 10 pm	12	16%	131	25%	

Bold means less than 0.05, *means significant values.

In addition, there was a significant association between BMI and type of diet (*p* = 0.005) and low carbohydrate or low-fat diet (*p* = 0.004). We also performed logistic regression to assess the probability of different dietary plans with demographic variables and eating habits. Our data showed that participants between 18 and 64 years old are 9–13 times more likely to follow any dietary plan. However, the 30–44 age group was the highest compared to other age groups (*p* = 0.02). In addition, participants residing in the eastern region of Saudi Arabia are 3 times more likely to apply any dietary plan than other regions of Saudi Arabia. It was demonstrated that participants who would approach any diet plan would exercise from 1 to 2 times up to 4–6 times per week. Participants are more likely to avoid unbalanced meals if a dietary plan is considered (Table 11). It also appears the following any dietary plan and low carbohydrate and low fat are significantly correlated with the BMI (Supplementary Table S4).

**Table 11:** Logistic regression for following any diet.

	Value of *p*	OR	95% C.I. for OR	
			Lower	Upper
Age				
65+ years		1.00		
18–29 years	**0.04**	9.95	1.08	91.53
30–44 years	**0.02**	13.09	1.47	116.53
45–64 years	**0.047**	9.04	1.03	79.41
Region				
Western		1.00		
Eastern	**0.02**	3.07	1.23	7.66
Central	0.09	2.24	0.88	5.68
Suffer from any chronic disease				
No		1.00		
Yes	0.06	1.68	0.99	2.87
Exercise for 30 min or more				
Do not exercise at all		1.00		
Mild (1–2 times per month)	**0.001**	2.94	1.54	5.59
Moderate (2–3 times per week)	**<0.001**	4.55	2.41	8.61
Extreme (4–6 times per week)	**<0.001**	9.23	4.19	20.35
Eat a healthy balanced meal				
No		1.00		
Yes	**0.00**	5.23	2.12	12.90
Sometimes	**0.01**	3.28	1.35	7.96
Servings of fruits per week				
0		1.00		
1–3	0.37	1.40	0.67	2.94
4 or more	0.95	0.97	0.39	2.42
Servings of vegetables per week				
0		1.00		
1–3	0.05	0.39	0.15	1.02
4 or more	0.23	0.54	0.19	1.49
Eating from restaurants				
I do not eat from restaurants		1.00		
Monthly	0.97	1.02	0.33	3.17
Weekly	0.94	1.04	0.35	3.09
Daily	0.34	0.54	0.16	1.89
Number of meals per day				
1–2		1.00		
3+	0.42	1.90	0.40	9.04
Time of last meal				
After 10 pm		1.00		
Before 7 pm	0.14	1.75	0.83	3.66
7–10 pm	0.69	1.13	0.63	2.02
BMI	**0.02**	1.06	1.01	1.10

Variable (s) entered on step 1: Age, Region, Suffer from any chronic disease, How often do you exercise for 30 min or more?, Do you eat a healthy balanced meal (carbohydrates, protein, and fat)?, How many servings of fruits do you eat per week?, How many servings of vegetables do you eat per week?, How often do you eat from restaurants?, How many meal (s) do you eat per day?, What time is your last meal?, BMI. Bold means less than 0.05.

In contrast, the participants are five times more likely to exercise if they follow IF than those who do not follow any dietary restrictions but eight times more likely to sometimes have a balanced diet, have 1–2 meals a day, and have their last meal before 7 pm (Table 12).

**Table 12:** Logistic regression for following intermittent fasting.

	Value of *p*	OR	95% C.I. for OR	
			Lower	Upper
Age				
65+ years		1.00		
18–29 years	1.00	117667432.23	0.00	
30–44 years	1.00	201636584.91	0.00	
45–64 years	1.00	140998941.28	0.00	
Gender				
Female		1.00		
Male	0.22	0.65	0.33	1.30
Exercise for 30 min or more				
I do not exercise at all		1.00		
Mild (1–2 times per month)	**0.001**	5.28	1.92	14.51
Moderate (2–3 times per week)	**0.001**	5.48	1.97	15.26
Extreme (4–6 times per week)	**0.01**	5.65	1.51	21.22
Eat a healthy balanced meal				
No		1.00		
Yes	0.08	6.42	0.82	50.19
Sometimes	**0.04**	8.19	1.07	62.57
Servings of fruits per week				
0		1.00		
1–3	0.30	1.70	0.62	4.69
4 or more	0.92	1.07	0.32	3.59
Eating from restaurants				
I do not eat from restaurants		1.00		
Monthly	0.87	0.90	0.24	3.31
Weekly	0.39	0.58	0.16	2.04
Daily	0.31	0.45	0.09	2.13
Number of meals per day				
3+		1.00		
1 to 2	**0.01**	2.37	1.23	4.59
Time of last meal				
After 10 pm		1.00		
Before 7 pm	**0.04**	3.13	1.07	9.18
7–10 pm	0.26	1.72	0.67	4.42

Variable (s) entered on step 1: Age, Gender, How often do you exercise for 30 min or more?, Do you eat a healthy balanced meal (carbohydrates, protein, and fat)?, How many servings of fruits do you eat per week?, How often do you eat from restaurants?, How many meal (s) do you eat per day?, What time is your last meal?. Bold means less than 0.05.

The logistic regression also shows that participants following a low-carbohydrate or Low-fat diet are expected to exercise 2–3 times or 406 times per week. They will be more likely to follow balanced meals (Table 13).

**Table 13:** Logistic regression for following low carb or low fat diet.

	Value of *p*	OR	95% C.I. for OR	
			Lower	Upper
Age				
65+ years		1.00		
18–29 years	>0.999	452012258.43	0.00	–
30–44 years	>0.999	391747371.45	0.00	–
45–64 years	>0.999	398220508.19	0.00	–
Suffer from any chronic disease				
No		1.00		
Yes	0.18	1.54	0.82	2.89
Exercise for 30 min or more				
I do not exercise at all		1.00		
Mild (1–2 times per month)	0.95	1.03	0.43	2.48
Moderate (2–3 times per week)	**0.01**	2.93	1.35	6.35
Extreme (4–6 times per week)	**<0.001**	5.15	2.13	12.45
Eat a healthy balanced meal				
No		1.00		
Yes	**0.01**	5.95	1.71	20.77
Sometimes	0.11	2.78	0.79	9.81
Servings of fruits per week				
0		1.00		
1–3	0.80	1.13	0.44	2.92
4 or more	0.61	1.34	0.44	4.08
Servings of vegetables per week				
0		1.00		
1–3	0.93	1.06	0.27	4.19
4 or more	0.71	1.31	0.32	5.45
BMI	**0.004**	1.08	1.02	1.14

Variable (s) entered on step 1: Age, Suffer from any chronic disease, How often do you exercise for 30 min or more? Do you eat a healthy balanced meal (carbohydrates, protein, and fat)?, How many servings of fruits do you eat per week?, How many servings of vegetables do you eat per week?, BMI. Bold means less than 0.05.

## Discussion

Overweight and obesity represent global health issues affecting 1.9 billion and more than 650 million people worldwide, respectively (25, 26). This health issue increases the risk of developing high blood pressure, dyslipidemia, diabetes, and other conditions, putting a substantial economic burden on many countries (27, 28). A sedentary lifestyle and consumption of fast food play a role in weight gain (29), and an increase in BMI above 40 kg/m2 would reduce life expectancy up to 20 years (27).

Bariatric surgeries are commonly performed in patients with a BMI greater than or equal to 40 kg/m^2^ or 35 mg/m^2^ with weight-related comorbidities (30). Some medications also help reduce weight by increasing metabolism; however, following dietary plans such as Ketogenic, intermittent fasting, a gluten-free diet, and a calorie restriction diet, alongside maintaining a healthy lifestyle, is suggested to complement these medications (31, 32).

This study provided insights into the dietary trends and eating habits of 608 individuals in Saudi Arabia. The demographic data showed that most participants were females. The high number of females interested could reflect their obsession with social norms than males and their self-consciousness about their figure (33, 34). In addition, the high responses observed from the eastern followed by the central regions of Saudi Arabia. This could be related to the higher BMI than 30 reported recently in these regions (7).

Obesity and overweight are major health concerns in Saudi Arabia that affect 20% of the population (6). However, the participants generally displayed a satisfactory level of awareness about maintaining a healthy lifestyle despite 75% of them not adhering to any specific dietary plan. The remainder followed either KD, IF, low carbohydrates, or GFD. Each of the dietary strategies serves a vital role in weight loss and health index. For example, the KD shows therapeutic value for obesity, polycystic ovarian syndrome, and cardiovascular diseases (35). The IF has also been reported to reduce body weight and improve dyslipidemia and blood pressure (36). This includes religious fasting named Ramadan IF, which has shown a protective role against inflammatory and oxidative stress markers (37). However, it is still unclear whether IF is beneficial for weight loss over a long period (38).

Additionally, a low-carbohydrate diet reduces cardiovascular risk factors in the short term, but further research is needed to determine long-term benefits (39). Although it is an efficient approach for weight loss, it is has been reported that many individuals quit as they find it difficult to adhere to this dietary plan (40). In this study, participants were advised to follow a gluten-free diet either for Celiac disease (CD) or food/skin allergy management. Despite the benefits of GFD, it could contribute to nutritional deficiencies such as vitamins, calcium, iron, and fiber (41). Also, adherence to a GFD is even more difficult due to the high cost of gluten-free products (42).

Furthermore, respondents were more likely to choose the IF as the dieting program in the first place, followed by the low-fat diet. People who decided those dieting programs would exercise (moderate to extreme) and observe healthy, balanced food. Not everyone would respond similarly to a specific diet because of metabolism variability between individuals (43).

Internet use has been shown to promote a healthy diet by increasing the intake of fruits, vegetables, and eggs and decreasing salt and oil intake (44). Our survey showed that the internet was the primary tool by which responders learned about these dietary schemes.

The timing of the last meal (after 7 p.m.) was previously associated with weight gain, dyslipidemia, high glucose levels, and metabolic syndrome and was frequently observed in obese individuals (45, 46). Data showed that most respondents would have their last meal after 7 p.m., highlighting the urge to increase awareness on the risk of obesity, hyperglycemia and metabolic syndromes (47, 48).

Most weight loss studies last at least 3 months or more (43). In most dietary types, participants following any diet involved in this study failed to continue for more than 3 months. The metabolism variability between individuals in response to similar food could explain why individuals would respond differently to a specific diet (43). This suggests that a personalized diet program might help sustain their dietary regimen. A recent study on personalized diet was suggested to aid in weight loss (49). Mithbavka et al. (50) presented an application for customized diet regimens, meeting the requirements for healthy food, depending on the user’s dietary preferences, and adapted to their health status (51).

Dietary guidelines for recommended healthy and balanced meals were released by the Saudi Ministry of Health in 2012. A study showed that the Saudi population needs to follow the guidelines for a healthy diet (52). These findings emphasize the need to increase awareness, as obesity and overweight are alarming issues affecting the Saudi population.

There are several limitations of this study. The cross-sectional nature creates temporal bias and limits any causal inferences. Moreover, selection bias due to convenience sampling is highly possible, limiting our study’s generalizability. However, this study is novel and bridges the gap in the literature due to the limited studies about dietary trends in Saudi Arabia.

Obesity remains a challenge in Saudi Arabia. Adherence to dietary regimes can help in controlling obesity. Increasing the public’s awareness of trending diets can aid in healthy lifestyles. Implementing a healthy lifestyle among the Saudi population to prevent weight gain and help in weight loss becomes necessary. Different methods can carry this out; one that seems efficient is through advertisements on social media and websites of general interest. Increasing the price of high-sugar or high-fat foods might help prevent weight gain and obesity.

Efforts to combat obesity in Saudi Arabia and globally require a multi-faceted approach, including promoting healthy eating habits, increasing physical activity, and raising awareness about the health risks associated with obesity. Public health interventions, community engagement, and policy changes are essential components of addressing the obesity epidemic in the country.

## Data availability statement

The original contributions presented in the study are included in the article/Supplementary material, further inquiries can be directed to the corresponding author.

## Ethics statement

The studies involving humans were approved by King Abdullah International Medical Research Center. The studies were conducted in accordance with the local legislation and institutional requirements. The participants provided their written informed consent to participate in this study.

## Author contributions

NoA: Writing – original draft, Writing – review & editing, Conceptualization, Data curation, Formal analysis. NaA: Data curation, Formal analysis, Investigation, Methodology, Writing – original draft, Writing – review & editing. ShA: Formal analysis, Writing – review & editing. MK: Writing – original draft, Writing – review & editing. RT: Data curation, Writing – original draft, Writing – review & editing. ME: Data curation, Writing – original draft. RW: Writing – original draft, Writing – review & editing. SaA: Writing – original draft, Writing – review & editing, Data curation. MR: Data curation, Formal analysis, Writing – review & editing. AO: Data curation, Formal analysis, Writing – review & editing. SiA: Formal analysis, Writing – original draft, Writing – review & editing. RS: Conceptualization, Data curation, Writing – original draft, Writing – review & editing. AA-B: Conceptualization, Data curation, Writing – original draft.
