# Supplementary material for: Dietary trends and obesity in Saudi Arabia
Source: Front Public Health. 2024 Jan 11;11:1326418. doi: 10.3389/fpubh.2023.1326418 (PMC10808649; doi:10.3389/fpubh.2023.1326418)
Supplement: Supplementary file 1 [file Table_1.docx]

|  |  | Follow any diet | | | |  |
| --- | --- | --- | --- | --- | --- | --- |
|  |  | Yes | | No | |  |
|  |  | n | % | n | % | p-value |
| Age | 18-29 years | 45 | 19% | 188 | 81% |  |
|  | 30-44 years | 61 | 30% | 140 | 70% | **0.008*** |
|  | 45-64 years | 45 | 29% | 111 | 71% |  |
|  | 65+ years | 1 | 6% | 17 | 94% |  |
| Gender | Male | 45 | 22% | 160 | 78% | 0.22 |
|  | Female | 107 | 27% | 296 | 73% |  |
| Nationality | Saudi | 118 | 26% | 344 | 74% | 0.58 |
|  | Non-Saudi | 34 | 23% | 112 | 77% |  |
| Marital status | Single | 49 | 21% | 183 | 79% |  |
|  | Married | 96 | 27% | 255 | 73% | 0.22 |
|  | Divorced / Widowed | 7 | 28% | 18 | 72% |  |
| Employment status | Employed | 61 | 26% | 178 | 74% |  |
|  | Non-employed | 39 | 29% | 96 | 71% | 0.68 |
|  | Retired | 15 | 26% | 43 | 74% |  |
|  | Self-employed | 5 | 26% | 14 | 74% |  |
|  | Student living with family | 26 | 20% | 106 | 80% |  |
|  | Student living away from home | 6 | 24% | 19 | 76% |  |
| Education level | High school or less | 26 | 20% | 104 | 80% |  |
|  | Bachelor's degree / Diploma | 105 | 26% | 296 | 74% | 0.33 |
|  | Higher degree (Masters, PhD or equivalent) | 21 | 27% | 56 | 73% |  |
| Income | I do not have an income | 51 | 26% | 144 | 74% |  |
|  | <10,000 SR | 26 | 20% | 103 | 80% |  |
|  | 10,000 to <20,000 SR | 31 | 28% | 81 | 72% | 0.68 |
|  | 20,000+ SR | 19 | 24% | 59 | 76% |  |
|  | I prefer not to say | 25 | 27% | 69 | 73% |  |
| Region | Eastern | 78 | 29% | 194 | 71% |  |
|  | Western | 9 | 15% | 51 | 85% | 0.07 |
|  | Central | 65 | 24% | 205 | 76% |  |
| Suffer from any chronic disease | No | 106 | 23% | 365 | 77% | **0.008*** |
|  | Yes | 46 | 34% | 91 | 66% |  |

**Table S1. Association of demographic variables with following any diet.**

|  |  | **Intermittent fasting diet** | | | |  |
| --- | --- | --- | --- | --- | --- | --- |
|  |  | Yes | | No | |  |
|  |  | n | % | n | % | p-value |
| Age (years) | 18-29 years | 17 | 7% | 216 | 93% | 0.12 |
|  | 30-44 years | 26 | 13% | 175 | 87% |  |
|  | 45-64 years | 16 | 10% | 140 | 90% |  |
|  | 65+ years | 0 | 0% | 18 | 100% |  |
| Gender | Male | 14 | 7% | 191 | 93% | 0.09 |
|  | Female | 45 | 11% | 358 | 89% |  |
| Nationality | Saudi | 47 | 10% | 415 | 90% | 0.49 |
|  | Non-Saudi | 12 | 8% | 134 | 92% |  |
| Marital status | Single | 19 | 8% | 213 | 92% | 0.39 |
|  | Married | 36 | 10% | 315 | 90% |  |
|  | Divorced/Widowed | 4 | 16% | 21 | 84% |  |
| Employment status | Employed | 24 | 10% | 215 | 90% | 0.90 |
|  | Non-employed | 16 | 12% | 119 | 88% |  |
|  | Retired | 5 | 9% | 53 | 91% |  |
|  | Self-employed | 1 | 5% | 18 | 95% |  |
|  | Student living with family | 11 | 8% | 121 | 92% |  |
|  | Student living away from home | 2 | 8% | 23 | 92% |  |
| Education level | High school or less | 8 | 6% | 122 | 94% | 0.23 |
|  | Bachelor's degree / Diploma | 41 | 10% | 360 | 90% |  |
|  | Higher degree (Masters, PhD or equivalent) | 10 | 13% | 67 | 87% |  |
| Income (SAR) | I do not have an income | 20 | 10% | 175 | 90% | 0.97 |
|  | <10,000 SR | 11 | 9% | 118 | 91% |  |
|  | 10,000 to <20,000 SR | 12 | 11% | 100 | 89% |  |
|  | 20,000+ SR | 8 | 10% | 70 | 90% |  |
|  | I prefer not to say | 8 | 9% | 86 | 91% |  |
| Region of Saudi Arabia | Eastern | 32 | 12% | 240 | 88% | 0.22 |
|  | Western | 3 | 5% | 57 | 95% |  |
|  | Central | 24 | 9% | 246 | 91% |  |
| Any chronic disease | No | 45 | 10% | 426 | 90% | 0.82 |
|  | Yes | 14 | 10% | 123 | 90% |  |

**Table S2. Association of demographic variables with following IF diet.**

|  |  | **Low Carb or Low-Fat diet** | | | |  |
| --- | --- | --- | --- | --- | --- | --- |
|  |  | Yes | | No | |  |
|  |  | n | % | n | % | p-value |
| Age (years) | 18-29 years | 24 | 10% | 209 | 90% | 0.11 |
|  | 30-44 years | 25 | 12% | 176 | 88% |  |
|  | 45-64 years | 26 | 17% | 130 | 83% |  |
|  | 65+ years | 0 | 0% | 18 | 100% |  |
| Gender | Male | 26 | 13% | 179 | 87% | 0.85 |
|  | Female | 49 | 12% | 354 | 88% |  |
| Nationality | Saudi | 58 | 13% | 404 | 87% | 0.77 |
|  | Non-Saudi | 17 | 12% | 129 | 88% |  |
| Marital status | Single | 25 | 11% | 207 | 89% | 0.46 |
|  | Married | 48 | 14% | 303 | 86% |  |
|  | Divorced/Widowed | 2 | 8% | 23 | 92% |  |
| Employment status | Employed | 29 | 12% | 210 | 88% | 0.64 |
|  | Non-employed | 19 | 14% | 116 | 86% |  |
|  | Retired | 9 | 16% | 49 | 84% |  |
|  | Self-employed | 3 | 16% | 16 | 84% |  |
|  | Student living with family | 11 | 8% | 121 | 92% |  |
|  | Student living away from home | 4 | 16% | 21 | 84% |  |
| Education level | High school or less | 12 | 9% | 118 | 91% | 0.44 |
|  | Bachelor's degree / Diploma | 54 | 13% | 347 | 87% |  |
|  | Higher degree (Masters, PhD or equivalent) | 9 | 12% | 68 | 88% |  |
| Income (SAR) | I do not have an income | 25 | 13% | 170 | 87% | 0.81 |
|  | <10,000 SR | 13 | 10% | 116 | 90% |  |
|  | 10,000 to <20,000 SR | 17 | 15% | 95 | 85% |  |
|  | 20,000+ SR | 9 | 12% | 69 | 88% |  |
|  | I prefer not to say | 11 | 12% | 83 | 88% |  |
| Region of Saudi Arabia | Eastern | 39 | 14% | 233 | 86% | 0.14 |
|  | Western | 3 | 5% | 57 | 95% |  |
|  | Central | 33 | 12% | 237 | 88% |  |
| Any chronic disease | No | 50 | 11% | 421 | 89% | **.017^*^** |
|  | Yes | 25 | 18% | 112 | 82% |  |

**Table S3. Association of demographic variables with following low carb or low-fat diet.**

|  |  | **Body Mass Index (kg/m2)** | | |  |
| --- | --- | --- | --- | --- | --- |
|  |  | n | Mean | sd | **p-value** |
| **Follow any diet** | Yes | 148 | 27.76 | 5.11 | **0.005** |
|  | No | 449 | 26.30 | 5.51 |  |
| **Follow Intermittent fasting diet** | Yes | 57 | 27.03 | 4.68 | 0.59 |
|  | No | 540 | 26.62 | 5.52 |  |
| **Follow Low Carb or Low Fat diet** | Yes | 73 | 28.38 | 4.78 | **0.004** |
|  | No | 524 | 26.42 | 5.49 |  |

**Table S4. Association of Body Mass Index with different types of diet.**
